# Efficacy and safety of adding rivaroxaban to the anti-platelet regimen in patients with coronary artery disease: a systematic review and meta-analysis of randomized controlled trials

**DOI:** 10.1186/s40360-018-0209-2

**Published:** 2018-05-02

**Authors:** Jun Yuan

**Affiliations:** Department of Cardiology, The People’s Hospital of Guangxi Zhuang Autonomous Region, Nanning, 530021 Guangxi China

**Keywords:** Rivaroxaban, Coronary artery disease, Dual anti-platelet therapy, Stent thrombosis, Minor bleeding, Major bleeding

## Abstract

**Background:**

Rivaroxaban, a direct factor Xa inhibitor, has seldom been used in patients with coronary artery disease. In this analysis, we aimed to systematically compare the efficacy and safety of rivaroxaban in addition to the anti-platelet regimen in patients with coronary artery disease.

**Methods:**

Online databases (MEDLINE, EMBASE, Cochrane database, www.ClinicalTrials.gov and Google scholar were searched for randomized controlled trials which were exclusively based on patients with coronary artery disease; and which compared efficacy (cardiovascular outcomes) and safety (bleeding outcomes) outcomes with the addition of rivaroxaban to the other anti-platelet agents. Analysis was carried out by the RevMan 5.3 software whereby odds ratios (OR) and 95% confidence intervals (CI) were generated following data input.

**Results:**

Four trials with a total number of 40,148 patients were included (23,231 participants were treated with rivaroxaban whereas 16,919 participants were treated with placebo) in this analysis. Patients’ enrollment period varied from years 2006 to 2016. The current results showed addition of rivaroxaban to significantly lower composite endpoints (OR: 0.81, 95% CI: 0.74–0.88; *P* = 0.00001). In addition, all-cause death, cardiac death, myocardial infarction, and stent thrombosis were also significantly reduced (OR: 0.82, 95% CI: 0.72–0.92; *P* = 0.0009), (OR: 0.80, 95% CI: 0.69–0.92; *P* = 0.002), (OR: 0.87, 95% CI: 0.77–0.98; *P* = 0.03) and (OR: 0.73, 95% CI: 0.55–0.97; *P* = 0.03) respectively. However, stroke was not significantly different.

However, TIMI defined minor and major bleeding were significantly higher with rivaroxaban (OR: 2.27, 95% CI: 1.47–3.49; *P* = 0.0002) and (OR: 3.44, 95% CI: 1.13–10.52; *P* = 0.03) respectively. In addition, intracranial hemorrhage and bleeding which was defined according to the International Society on Thrombosis and Hemostasis criteria were also significantly higher with rivaroxaban (OR: 1.63, 95% CI: 1.04–2.56; *P* = 0.03) and (OR: 1.80, 95% CI: 1.45–2.22; *P* = 0.00001) respectively. Nevertheless, fatal bleeding was not significantly different.

**Conclusions:**

Addition of rivaroxaban to the anti-platelet regimen was effective in patients with coronary artery disease, but the safety outcomes were doubtful. Further future trials will be able to completely solve this issue.

## Background

Rivaroxaban, a direct factor Xa inhibitor, has seldom been used in patients with stable coronary artery disease. Even if recently published studies have already compared rivaroxaban with dabigatran in patients with atrial fibrillation [1], the use of rivaroxaban in patients with coronary artery disease or following percutaneous coronary intervention (PCI) is still under study.

The PIONEER-AF-PCI Trial which consists of 2100 participants, is an exploratory, open-labelled, randomized, multi-center clinical study which is being carried out to assess the safety of rivaroxaban and vitamin K antagonist treatment strategy in patients with non-valvular atrial fibrillation who will require PCI [2]. Also, the RT-AF trial which is also an open-labelled study enrolling patients with non-valvular atrial fibrillation who will require coronary stenting, and who will require either triple therapy (warfarin, clopidogrel and aspirin) or dual therapy (rivaroxaban and ticagrelor) following intervention, is still under investigation [3] whereas the COMMANDER HF Trial, which is an International prospective, randomized, doubled-blind, placebo-controlled study comparing rivaroxaban with placebo in patients with heart failure and coronary artery disease, has not been published yet [4].

Plaque rupture and thrombosis are major concerns in patients with atherosclerosis. Dual antiplatelet therapy (DAPT) with aspirin and clopidogrel is considered the standard antiplatelet regimen especially after coronary intervention with drug eluting stents [5]. However, a more potent regimen was urgently needed due to limitations of these usual anti-platelet agents.

Recently, Bundhun et al. showed the addition of cilostazol to the standard DAPT in patients with acute coronary syndrome to be effective [6]. However, the unwanted safety outcomes which persisted with the use of cilostazol resulted in drug discontinuation indicating that other more effective agents would be required.

In this analysis, we aimed to systematically compare the efficacy and safety of rivaroxaban in addition to the anti-platelet regimen in patients with coronary artery disease.

## Methods

### Searched databases

The following databases were searched for randomized controlled trials:

1. MEDLINE/PubMed;
2. EMBASE (www.sciencedirect.com);
3. Cochrane database;
4. www.ClinicalTrials.gov;
5. Google scholar.

### Searched terms

The online databases were carefully searched for English language publications (from November to December 2017). Publications included the titles with their associated abstract or full-text articles.

Keywords which were used in the search process included:

1. Rivaroxaban and coronary artery disease;
2. Rivaroxaban and percutaneous coronary intervention;
3. Rivaroxaban and dual anti-platelet therapy;
4. Rivaroxaban and aspirin and clopidogrel;
5. Xarelto and percutaneous coronary intervention;
6. Rivaroxaban and drug eluting stents.

### Inclusion and exclusion criteria

Studies were included based on the following criteria:

1. They were exclusively randomized controlled trials;
2. They were exclusively based on patients with coronary artery disease;
3. They compared outcomes which were observed with the addition of rivaroxaban to other anti-platelets;
4. They reported adverse cardiovascular (efficacy) and bleeding (safety) outcomes as their endpoints.

Studies were excluded if:

1. They were non-randomized controlled trials (observational cohorts, meta-analysis and systematic reviews, case studies);
2. They involved patients who were treated for other conditions (peripheral artery disease, non-valvular atrial fibrillation);
3. They did not report adverse cardiovascular or bleeding outcomes;
4. They were duplicated studies.

### Outcomes, definition and follow-ups

The outcomes (Table 1) which were assessed were as followed:

1. Composite endpoint: consisting of a combination of cardiac death, myocardial infarction, stroke and or stent thrombosis;
2. All-cause mortality;
3. Myocardial infarction (MI);
4. Cardiac death;
5. Stent thrombosis;
6. Stroke;
7. Thrombolysis in myocardial infarction (TIMI) defined minor and major bleedings [7];
8. Fatal bleeding: defined as severe life-threatening bleeding;
9. Intracranial hemorrhage;
10. Bleeding which was defined by the International Society on Thrombosis and Hemostasis (ISTH) [8].

**Table 1:** Outcomes which were assessed

Trials	Outcomes reported	Follow-up periods	Drugs which were used	Dosage of rivaroxaban
ATLAS-ACS 2 TIMI 51 [11]	Cardiac death + stroke + MI (composite endpoint), cardiac death, MI, stroke, all-cause death, ST, TIMI minor and major bleeding, Intracranial hemorrhage, fatal bleeding	13 to 31 months	Rivaroxaban + DAPT versus DAPT (aspirin + clopidogrel)	2.5 mg or 5 mg twice daily
GEMINI-ACS-1 [12]	Cardiac death + stroke + MI + ST (composite endpoint), cardiac death, MI, stroke, all-cause death, ST (definite + probable), TIMI minor and major bleeding, fatal bleeding, intracranial hemorrhage, ISTH major bleeding	1 year	Ribaroxaban + clopidogrel/ticagrelor versus aspirin + clopidogrel/ticagrelor	2.5 mg twice daily
COMPASS [13]	Cardiac death + stroke + MI (composite endpoint), all-cause death, cardiac death, stroke, MI, fatal bleeding, intracranial hemorrhage, major bleeding according to ISTH criteria	23 months	Rivaroxaban + aspirin versus aspirin alone	2.5 mg or 5 mg twice daily
ATLAS-ACS-TIMI 46 [14]	TIMI minor and major bleeding, death + MI + stroke (composite endpoint)	6 months	Rivaroxaban + DAPT or aspirin versus DAPT (aspirin + clopidogrel)	5 to 20 mg once daily or the same total dose given twice daily

*MI* myocardial infarction, *ST* stent thrombosis, *TIMI* thrombolysis in myocardial infarction, *ISTH* International Society on Thrombosis and Hemostasis, *DAPT* dual anti-platelet therapy

The follow-up periods ranged from 6 months to 31 months as shown in Table 1.

### Data extraction and review

The following data were extracted by the author Jun Yuan:

1. Type of study;
2. Time period of patients’ enrollment;
3. Total number of patients in the experimental and control groups respectively;
4. The methodological quality of the trials;
5. The baseline features of the participants;
6. The adverse cardiovascular and bleeding outcomes;
7. The total number of events which were reported in each subgroup.

Data were also searched, extracted, screened and checked by Dr. Guang Ma Xu whom we have acknowledged at the end of the manuscript.

The review protocol was not prospectively registered.

The methodological quality of each trial was assessed based on the criteria which has been suggested/recommended by the Cochrane Collaboration [9] whereby scores were given depending on the risk of bias which was reported, and the highest which was 12 points indicated lowest risk of bias.

The PRISMA Guideline was followed [10].

### Statistical analysis

This analysis was carried out by the most common software which has been used for decades to carry out meta-analyses: The RevMan 5.3 software. Following data input through the software, odds ratios (OR) with 95% confidence intervals (CI) were generated.

Heterogeneity was assessed by:

1. The Q statistic test whereby a *P* value less or equal to 0.05 was considered as statistically significant.
2. The I^2^ statistical test whereby heterogeneity was increased with increasing I^2^.

Two statistical effect models were applied based on the value of I^2^:

If I^2^ was less than 50%, a fixed effects model was used. However, if I^2^ was > 50%, a random effects model was used.

Sensitivity analysis was carried out by an exclusion method whereby each trial was excluded one by one and a new analysis was generated each time to observe any significant change in the results.

In addition, publication bias was assessed through the funnel plot which was generated by the software.

## Results

### Searched outcomes

Search which was carried out from online (electronic) databases resulted in a total number of 298 publications. A pre-assessment was carried out whereby 259 articles were excluded since they were not related to the scope of this research. Thirty-nine (39) full-text articles were assessed for eligibility. Further elimination was carried out based on the following criteria:

1. They were non-randomized controlled trials (3);
2. They were not exclusively based on patients with coronary artery disease (14);
3. They were repeated studies (18).

**Fig. 1:**
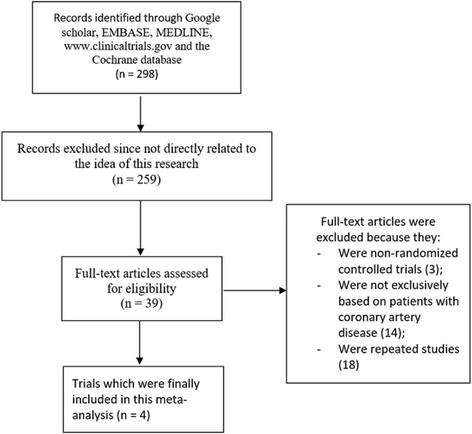
Flow diagram representing the study selection

Finally, only 4 trials [11–14] were selected for this meta-analysis (Fig. 1).

### General features of the trials

The general features of the trials have been listed in Table 2. A total number of 40, 148 patients were included in this analysis (23,231 participants were treated with rivaroxaban whereas 16,919 participants were treated with placebo). Patients’ enrollment period varied from the year 2006 to the year 2016 as shown in Table 2.

**Table 2:** General features of the trials which were included

Trials	No of patients treated by rivaroxaban (n)	No of patients in the placebo group (n)	Time period of patients’ enrollment	Bias risk score
ATLAS-ACS 2 TIMI 51	10,229	5113	2008–2011	10
GEMINI-ACS-1	1519	1518	2015–2016	10
COMPASS	9152	9126	2013–2016	10
ATLAS-ACS-TIMI 46	2331	1160	2006–2008	10
Total no of patients (n)	23,231	16,917		

After a careful assessment of the risk of bias, all the four trials were allotted a score of 10 out of 12, representing a low risk of bias.

### Baseline features of the participants

The baseline features of the participants have been listed in Table 3. The average age varied from 57.2 to 68.2 years. Majority of the participants were male patients. The percentage of patients with high blood pressure, dyslipidemia, diabetes mellitus and a smoking history has been represented in Table 3. According to the data given, no significant difference was observed in patients who were assigned to the experimental or the control groups.

**Table 3:** Baseline features of the participants

Trials	Age (years)	Males (%)	HT (%)	Ds (%)	DM (%)	Cs (%)
	R/placebo	R/placebo	R/placebo	R/placebo	R/placebo	R/placebo
ATLAS-ACS 2 TIMI 51	61.9/61.5	74.6/75.0	67.4/67.5	48.7/48.2	32.1/31.8	–
GEMINI-ACS-1	62.0/63.0	75.0/75.0	71.0/75.0	56.0/56.0	29.0/30.0	32.0/34.0
COMPASS	68.3/68.2	67.5/68.2	75.5/75.4	–	37.7/38.1	3.80/3.70
ATLAS-ACS-TIMI 46	57.2/57.8	77.6/76.3	57.4/56.9	44.4/43.6	19.4/19.1	61.9/62.6

*R* rivaroxaban group, *HT* hypertension, *Ds* dyslipidemia, *DM* diabetes mellitus, *Cs* current smoker

### Main results of this analysis

The main results of this analysis were shown in Table 4.

**Table 4:** Results of this analysis

Outcomes which were assessed	OR with 95% CI	*P* value	I^2^ (%)
Composite endpoint	0.81 [0.74–0.88]	0.00001	19
All-cause mortality	0.82 [0.72–0.92]	0.0009	0
Cardiac death	0.80 [0.69–0.92]	0.002	0
Myocardial infarction	0.87 [0.77–0.98]	0.03	10
Stent thrombosis	0.73 [0.55–0.97]	0.03	28
Stroke	0.77 [0.43–1.38]	0.38	81
TIMI defined minor bleeding	2.27 [1.47–3.49]	0.0002	0
TIMI defined major bleeding	3.44 [1.13–10.52]	0.03	74
Intracranial bleeding	1.63 [1.04–2.56]	0.03	44
Fatal bleeding	1.19 [0.74–1.91]	0.48	0
ISTH bleeding	1.80 [1.45–2.22]	0.00001	0

*TIMI* thrombolysis in myocardial infarction, *ISTH* International Society on Thrombosis and Hemostasis, *OR* odds ratios, *CI* confidence intervals

**Fig. 2:**
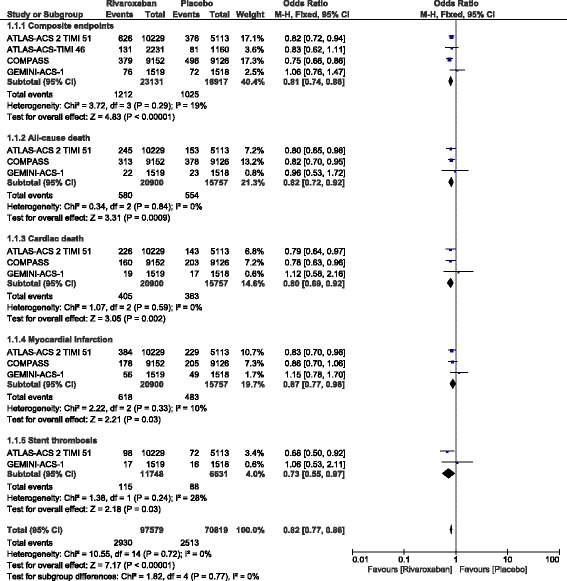
Outcomes assessing efficacy with the addition of rivaroxaban in patients with coronary artery disease

Results of this analysis showed that addition of rivaroxaban to the anti-platelet regimen significantly lowered composite endpoints (OR: 0.81, 95% CI: 0.74–0.88; *P* = 0.00001, I^2^ = 19%). In addition, all-cause death, cardiac death, MI, and stent thrombosis were also significantly reduced (OR: 0.82, 95% CI: 0.72–0.92; *P* = 0.0009, I^2^ = 0%), (OR: 0.80, 95% CI: 0.69–0.92; *P* = 0.002, I^2^ = 0%), (OR: 0.87, 95% CI: 0.77–0.98; *P* = 0.03, I^2^ = 10%) and (OR: 0.73, 95% CI: 0.55–0.97; *P* = 0.03, I^2^ = 28%) respectively as shown in Fig. 2.

**Fig. 3:**
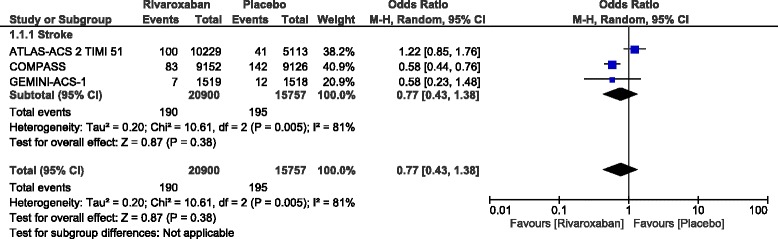
Stroke observed with the addition of rivaroxaban in patients with coronary artery disease

However, stroke was not significantly different (OR: 0.77, 95% CI: 0.43–1.38; *P* = 0.38) as shown in Fig. 3.

**Fig. 4:**
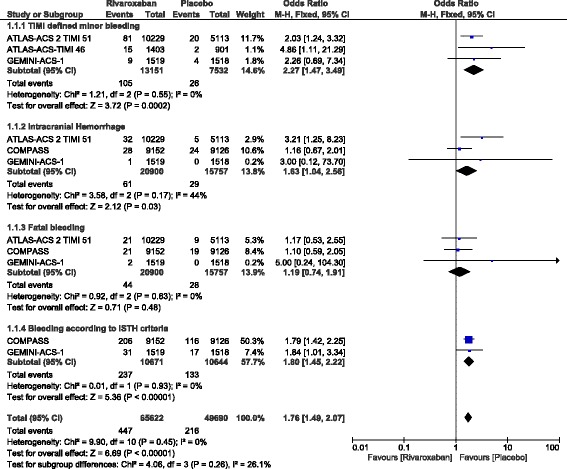
Safety outcomes with the addition of rivaroxaban in patients with coronary artery disease

**Fig. 5:**
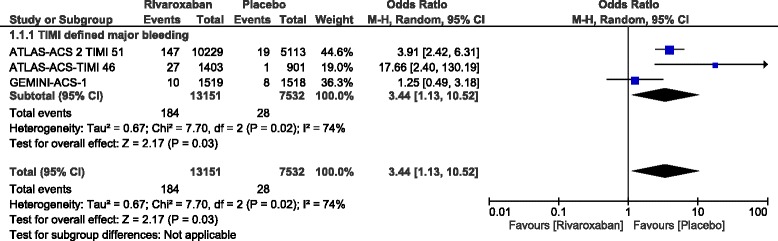
TIMI defined major bleeding observed with the addition of rivaroxaban in patients with coronary artery disease

Bleeding outcomes (safety outcomes) were also assessed. The current results showed TIMI defined minor and major bleeding to be significantly higher with rivaroxaban (OR: 2.27, 95% CI: 1.47–3.49; *P* = 0.0002, I^2^ = 0%) and (OR: 3.44, 95% CI: 1.13–10.52; *P* = 0.03, I^2^ = 74%) respectively as shown in Figs. 4 and 5.

Intracranial hemorrhage and bleeding which was defined according to the ISTH criteria were also significantly higher with rivaroxaban (OR: 1.63, 95% CI: 1.04–2.56; P = 0.03, I^2^ = 44%) and (OR: 1.80, 95% CI: 1.45–2.22; *P* = 0.00001, I^2^ = 0%) respectively. However, fatal bleeding was similarly manifested (OR: 1.19, 95% CI: 0.74–1.91; *P* = 0.48, I^2^ = 0%) as shown in Fig. 4.

### Sensitivity analysis and publication bias

**Fig. 6:**
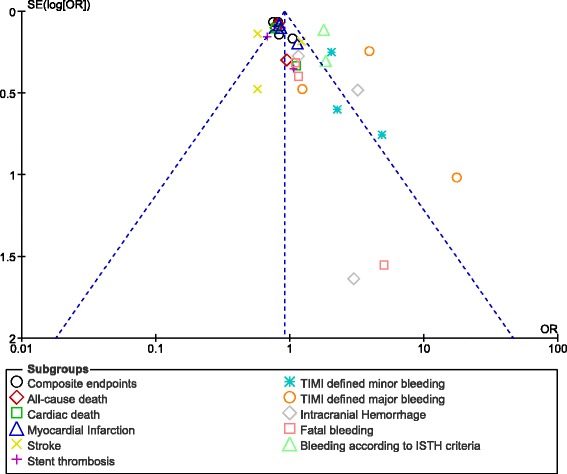
Funnel plot representing publication bias

Consistent results were obtained through sensitivity analyses. In addition, based on a visual assessment of the funnel plot, which was a better way to illustrate publication bias in an analysis including a small volume of studies, there was low to moderate evidence of publication bias across all the studies that compared the outcomes assessing efficacy and safety with the addition of rivaroxaban to the anti-platelet regimen in patients with coronary artery disease as shown in Fig. 6.

## Discussion

Antiplatelet and anti-thrombotic therapies are vital in patients with coronary artery disease especially after PCI. DAPT (aspirin and clopidogrel) has been used for decades in patients who were implanted with bare metal stents and drug eluting stents [15]. However, more potent drugs or adjuvants were still required to overcome the limitations observed with the commonly used anti-platelet agents.

Recently, Bundhun et al. showed addition of cilostazol to the standard DAPT to be associated with significantly lower adverse outcomes including revascularization [6]. However, cilostazol was associated with unwanted safety outcomes. This was the main reason for drug discontinuation among several of the participants who were randomized to take part in the study. For example, cilostazol was observed to be associated with more headache, diarrhea, skin rashes and tachycardia/palpitations [16] when compared to the other anti-platelet agents. In addition, cilostazol is apparently contraindicated in congestive heart failure [16]. Even if significance was not reached, cilostazol use in the triple anti-platelet regimen was associated with a higher risk of major and minor bleeding when compared to DAPT without cilostazol [17].

In this analysis, rivaroxaban was added to DAPT or to aspirin alone in patients with coronary artery disease, and the efficacy and safety outcomes were assessed in comparison to placebo. Current results showed significantly lower death, myocardial infarction, and stent thrombosis associated with the addition of rivaroxaban to the anti-platelet regimen. However, when the safety outcomes were assessed, TIMI defined minor and major bleedings, intracranial bleeding as well as bleeding which was defined according to the ISTH criteria were significantly increased. In other words, the addition of rivaroxaban to the antiplatelet drug regimen was effective, but the safety outcomes appeared critical.

In the X-PLORER Trial, whereby 111 participants were enrolled (October 2011 to March 2013), rivaroxaban was used during the invasive procedure in patients with stable coronary artery disease [18]. The authors found rivaroxaban to effectively suppress the activation of coagulation following stenting. TIMI defined minor and major bleedings as well as bleeding defined by the Academic Research Consortium (ARC) were not elevated during a follow up period of 30 days. Nevertheless, the X-PLORER Trial was different from our current analysis in terms of shorter follow-up period, and this direct factor Xa inhibitor was used only during the procedure.

Similar to this analysis, the ATLAS-ACS 2 TIMI 51 Trial showed a reduction in mortality and stent thrombosis with the addition of rivaroxaban along with DAPT following PCI in patients with acute coronary syndrome [19].

In contrast, the GEMINI-ACS-1 trial which consisted of 3037 participants showed rivaroxaban to exhibit similar significant bleeding to that of aspirin and clopidogrel [12]. However, it should be noted that the study had a shorter follow-up duration time period of 291 days and the total number of participants was also less in comparison to other studies. In addition, the participants only received 2.5 mg rivaroxaban twice daily in comparison to other studies whereby the dosage was doubled.

Another review even suggested substituting aspirin for rivaroxaban (2.5 mg twice daily) due to a similar bleeding risk [20]. However, since it was a phase II study, the authors specified that a larger phase III trial will be able to confirm their findings.

### Novelty

This research article is new in the following ways:

1. It is the first study to systematically analyze the addition of rivaroxaban to the anti-platelet regimen in patients with coronary artery disease;
2. This idea is new in clinical medicine, and is still being studied;
3. Only data which were obtained from randomized trials (good data) have been included;
4. A very low level of heterogeneity was observed among almost all the subgroups that assessed the efficacy and safety endpoints.

### Limitations

Limitations were as followed:

1. This analysis consisted of a limited number of trials and participants;
2. The placebo involved different drug regimens in different trials. Two trials involved DAPT in the placebo group. However, one trial involved only aspirin, and another one consisted only of clopidogrel or ticagrelor. This might have had an effect on the results;
3. Different trials had different follow-up periods which might have influenced the results;
4. Other bleeding outcomes such as bleeding defined by the academic research consortium, and GUSTO bleeding, could not be assessed because they were reported in only one study.
5. Dosage of rivaroxaban might have also influenced the results.

## Conclusions

Addition of rivaroxaban to the anti-platelet regimen was effective in patients with coronary artery disease, but the safety outcomes were doubtful. Further future trials will be able to completely solve this issue.

## Abbreviations

DAPT: Dual anti-platelet therapy
PCI: Percutaneous coronary intervention
RCT: Randomized controlled trials
TIMI: Thrombolysis in myocardial infarction
